# Detection of Driving Capability Degradation for Human-Machine Cooperative Driving

**DOI:** 10.3390/s20071968

**Published:** 2020-04-01

**Authors:** Feng Gao, Bo He, Yingdong He

**Affiliations:** 1 School of Automotive Engineering, Chongqing University, Chongqing 400044, China; 2 Department of Intelligent Vehicle, Chang’an Global Automobile Research Center, Chongqing 401133, China; hebo@changan.com.cn; 3 Mechanical Engineering, University of Michigan, Ann Arbor, MI 48109, USA; heyingd@umich.edu

**Keywords:** automatic driving, cooperative driving, driving capability, driving risk, driver state, driver model

## Abstract

Due to the limitation of current technologies and product costs, humans are still in the driving loop, especially for public traffic. One key problem of cooperative driving is determining the time when assistance is required by a driver. To overcome the disadvantage of the driver state-based detection algorithm, a new index called the correction ability of the driver is proposed, which is further combined with the driving risk to evaluate the driving capability. Based on this measurement, a degraded domain (DD) is further set up to detect the degradation of the driving capability. The log normal distribution is used to model the boundary of DD according to the bench test data, and an online algorithm is designed to update its parameter interactively to identify individual driving styles. The bench validation results show that the identification algorithm of the DD boundary converges finely and can reflect the individual driving characteristics. The proposed degradation detection algorithm can be used to determine the switching time from manual to automatic driving, and this DD-based cooperative driving system can drive the vehicle in a safe condition.

## 1. Introduction

With the growing number of vehicles, traffic safety has become a hot social concern. The driver error, caused by visual distraction, fatigue, etc., contributes to most traffic accidents [1,2]. In recent years, the world has been devoted to the development of automatic driving technologies because they can enhance the sense ability of risk, correct dangerous driving behavior and even control the vehicle completely without any intervention from humans [3,4]. However, due to the limitation of current technologies and product costs, the driverless car running in public traffic is still under research and humans will be in the driving loop for a long time [5,6]. Hence, the human-machine cooperative driving, which allows both machines and humans to pilot concurrently, has drawn much attention recently [7,8].

One key problem of cooperative driving is evaluating the current status of the driver, and the driver attentiveness is the most considered index [9]. There are mainly two types of indices for the evaluation of driver attentiveness, i.e., distraction and fatigue. A variety of measuring methods and instruments have been developed, which can be divided into direct and indirect approaches. The direct measurements usually use physiological signals or driver body behavior, e.g., electroencephalogram, electrocardiogram and face features, including the movement of the head and eyes [10,11]. Though the physiological information has a high accuracy, the extra measurement instrument causes intrusive interference, which has a negative impact on driving. Moreover, the acceptability and usability of such systems is also a problem. For instance, wearing glasses makes it difficult to locate the position of the eyes, and the intensity of illumination also influences the quality of images. Comparatively, the indirect ones only use such signals as the steering angle, time to collision, headway time, etc., which are measured by on-board sensors [11]. To evaluate the driver state correctly, some intelligent algorithms are adopted to process these signals for a better anti-interference performance, for example statistical analyses and machine learning methods [11,12,13].

With the aforementioned measuring technologies of the driver state, several cooperative driving systems have been proposed. Tran et al. designed a switching logic from manual driving to automatic mode by using the fatigue information of drivers [14]. In this study, both face features and steering operations are used. Compared with those using only one type of signal, the detection accuracy of fatigue is improved by multiple mode information combined with the learning classification algorithm. Pohl et al. designed a cooperative lane keeping assistance system according to the driver’s state of visual distraction, which is detected by the data of the head pose and eye movement [15]. To improve the robustness of detection and adaptability to more general traffic conditions, Enache et al. combined the body information with the steering torque to detect the driver’s distraction [16]. The developed assistance system can be applied to roads with different curvatures and running velocities. Since both visual distraction and fatigue increase the driving risk, Benloucif et al. defined a variable to evaluate these two types of driver states together [17]. Each driver state is described by a binary signal, i.e., eye-off-road or not and critical fatigue or not, respectively. 

These driver state based cooperative driving systems still have some disadvantages in practical applications. Even if we can get the accurate information of the driver state, the momentary behavior, such as checking the rearview mirror, may still cause a false judgement and lead to an intervention on the driving. Sentouh et al. have already been aware of this problem. A mandatory trigger delay of 3 s was suggested to prevent such false interventions when they developed a shared driving system [18]. Another disadvantage is that the driver state is not totally equal to the driving ability, that is to say an inattentive driver may still be able to deal with the driving task safely, especially when the driving difficulty is very low. On the contrary, an attentive driver is also likely to make a wrong operation under complicated traffic conditions [19]. For example, Liang et al. found that cognitive distraction may enhance the lateral control performance of drivers under some special conditions [20].

To comprehensively measure the driving capability and avoid unnecessary interventions on drivers, a new index called the correction ability of drivers is designed for the first time and is combined with the driving risk to form the evaluation space for the driving capability. Based on this two-dimensional evaluation space, a degraded domain (DD) is set up to detect the degradation of the driving capability. According to the simulated driving data, the log normal distribution is used to model the boundary of DD, and an online algorithm is designed for the estimation of its parameter to characterize the individual driving style. This new degradation detection method for cooperative driving is more accurate than that only using the driver state. The DD based cooperative driving system can reserve enough time for correction by the automatic driving system, and the transition process is smooth enough.

The rest of the paper is organized as follows: Section 2 introduces the fundamentals of the detection method for the driving capability; Section 3 establishes the model of driver behavior under normal conditions; in Section 4, the degraded domain to detect the degradation of the driving capability is designed; the proposed strategy is validated and analyzed by a bench test in Section 5 and applied to the cooperative driving system in Section 6; Section 7 concludes the paper. 

## 2. Detection Strategy of Driving Capability Degradation

**Figure 1:**
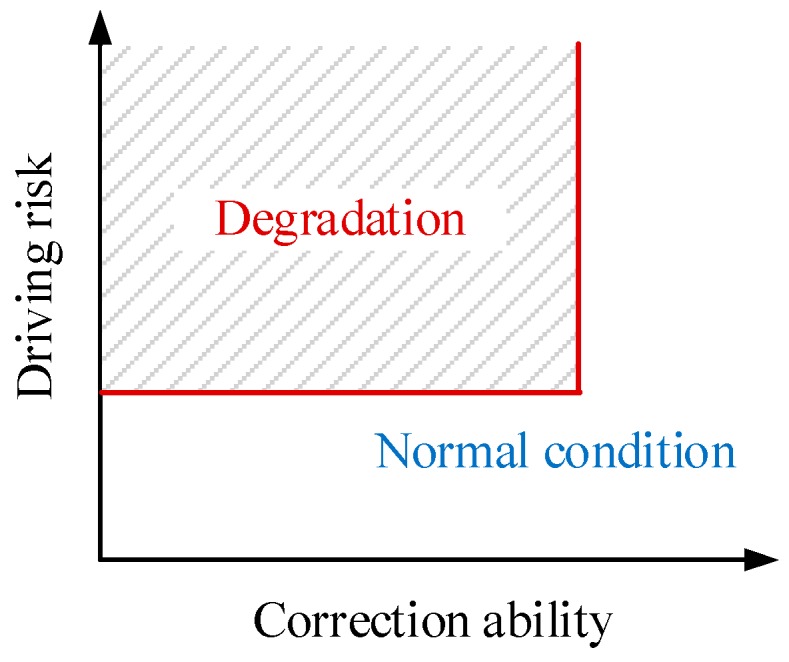
Two-dimensional evaluation space for the driving capability.

To overcome the disadvantages of direct methods, and considering the fact that the information of vehicles contains the data required to measure the driving capability since it is the ultimate embodiment of all drivers’ operations, only onboard signals are used in this study. A two-dimensional space, as shown in Figure 1, is proposed to evaluate the driving capability because: (1) For a cooperative driving system, it is important to ensure that the assistance is only provided when the driver requires it; otherwise, the intervention may cause a negative impact on driving; (2) Accordingly, the assistance is not necessary if the driver has an adequate ability to correct the vehicle state even when the driving risk is high.

Instead of the traditional driver state, a new index is designed, which is evaluated in a two-dimensional space, considering both the driving risk and correction ability of the driver. The former focuses on the collision risk among the ego-vehicle and other objects. The latter is for the evaluation of the driver’s ability in managing the vehicle in a risk-free state. Referring to [21], we have designed the following degradation detection logic for the driving capability:

(1) $$\{\begin{array}{cc} x_{d}\in \Omega , & \mathrm{Degradation}\\ \mathrm{Otherwise}, & \;\mathrm{Normal}\;\mathrm{driving} \end{array},\;\;x_{d}=\left[\begin{array}{c} \varepsilon_{r}\\ \varepsilon_{c} \end{array}\right]$$

where $x_{d}\in {\mathbb{R}}^{2}$ is the driving state, $\varepsilon_{r},\;\varepsilon_{c}$ are the driving risk and correction ability, and Ω is a set composed of all degraded driving states, called DD here, as shown by the shadow area in Figure 1. The details of the design of Ω can be found in Section 4. It should be noted that the longitudinal and lateral driving capability is dealt with separately, but the detection logic is the same as in Equation (1). This implies that even if the longitudinal driving performance of the driver is degraded, the steering may still be controlled by the driver finely. Compared with the driver state-based detection strategy, this new logic further considers the controllability of the driver to avoid unnecessary interventions.

### 2.1. Driving Risk

Compared with the evaluation of the correction ability that has been very little investigated, how to measure the driving risk has been widely studied to design such systems as the lane departure waning and forward collision warning systems [22,23]. In these studies, time-to-line crossing (TLC) [23] and time to collision (TTC) [23,24] are mainly used to determine when to activate the warning, and so TLC and TTC are used to measure the driving risk in this paper:

(2) $$\begin{array}{l} \mathrm{Lateral}:\;\mathrm{TLC}=\left(\Delta_{L}-0.5W\right)/\left(v_{s}\sin\varphi \right)\\ \;\mathrm{Longitudinal}:\;\mathrm{TTCL}=\Delta_{V}/D\; \end{array}$$

where $\Delta_{L}$ is the distance from the gravity center of the vehicle to the crossed line, W is the width of the vehicle, φ is the relative yaw angle to the road direction, $v_{s}$ is the velocity of the vehicle, $\Delta_{V}$ is the relative velocity and D is the distance. Here, TTCi is used instead of TTC to avoid D being divided by zero when $\Delta_{V}=0$.

### 2.2. Correction Ability

Few studies have looked into the evaluation of the driver’s correction ability. Being different from the driver state, which can be measured by direct [10] or indirect [11] methods, the correction ability is hard to measure because it characterizes the managing ability of the driver under abnormal conditions. The following hypotheses are assumed in this paper when designing the measurement index of the correction ability:

(1) A driver has his/her own desired input to the vehicle when faced with a specific driving condition;
(2) Additionally, when the driver has an adequate control capability, his/her actual input will not deviate from the desired one too much.

As a result, the deviation between the actual and desired ones is selected to measure the correction ability:

(3) $$\begin{array}{c} \mathrm{Lateral}:\;\epsilon_{cla}=\left|\delta_{a}-\delta_{m}\right|\\ \;\;\;\;\;\mathrm{Longitudinal}:\;\epsilon_{clo}=\left|a_{a}-a_{m}\right|\;\;\;\;\;\;\;\;\;\; \end{array}$$

where $\epsilon_{cla}$ and $\epsilon_{clo}$ are the lateral and longitudinal correction abilities, $\delta_{a}$ and $\delta_{m}$ are the actual and desired steering angles, and $a_{a}$ and $a_{m}$ are the actual and desired longitudinal accelerations respectively. 

## 3. Driver’s Desired Input Prediction

Various types of driver behavior models have been developed over decades. The earlier models treat the driver behavior as linear time-invariant dynamics. Then, the error compensation and preview strategy is adopted to describe the dynamical process of driving [25,26,27]. In practical traffic, driver behavior shows the characteristic of a strong randomness, nonlinearity and personalization. Machine learning algorithms have been adopted to achieve a better accuracy and adaptability [28], such as the Gauss Mixed and Hidden Markov Model (GMM-HMM) [24], Artificial Neural Network for Nonlinear Autoregressive Exogenous Process (ANN-NARX) [29], Deep Believe Network (DBN) [30], etc. Compared to others, NARX is a nonlinear autoregressive exogenous process containing an input delay, which leads to an extra ability to describe the reaction time of the driver. Furthermore, ANN-NARX is a NARX model using an artificial neural network (ANN) to fit its relationship, which can take the advantages of both NARX and ANN. Thus, ANN-NARX was selected in this paper to calculate the desired input of the driver.

### 3.1. Test Bench and Scenario

**Figure 2:**
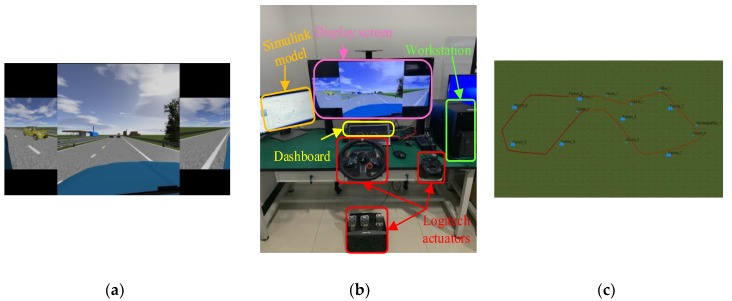
Test bench for the collection of data for model training and validation: (**a**) Display information to the driver; (**b**) Construction of the test bench; and (**c**) Trajectory of the driving scenario.

The driving data for training the driver model is required in order to cover relatively comprehensive conditions that take velocity, road geometry, traffic users and other factors into account. One way to do this is to use naturalistic driving data, which is recorded in real traffic and involves the real reaction of the driver. However, this is very time- and cost-consuming, and moreover the conditions are uncontrollable and noisy, which is bad for the convergence of the training process. A driver-in-the-loop simulator is constructed, as shown in Figure 2, to collect the data set [31], which is also used to validate the proposed transition strategy for consistency in Section 5. The traffic environment is simulated in real time by Prescan, which is a professional software to simulate and test automatic driving systems. The real time simulator MicroLab with the Automotive Simulation Model (ASM) running on it provides a vivid vehicle dynamical behavior.

**Figure 3:**
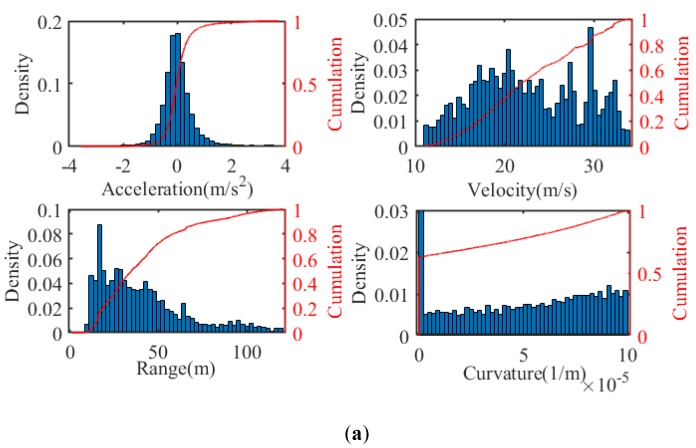

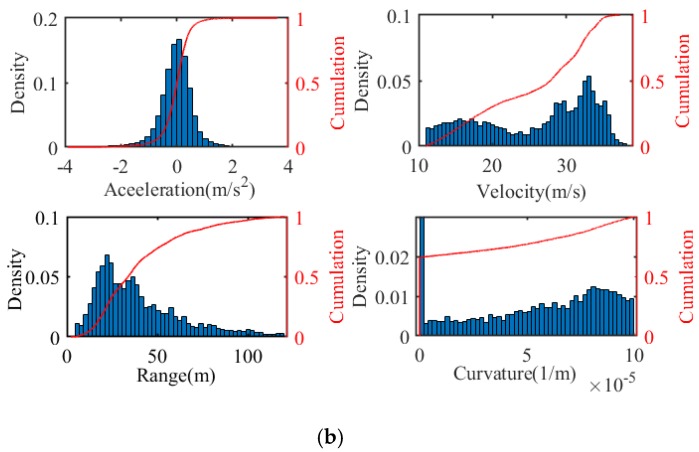
Statistical results of the driving data: (**a**) Driver 1; and (**b**) Driver 2.

The trajectory depicted in Figure 2c is about 70 km, which includes straight and bent roads with different curvature radiuses. Various types of vehicles, such as light passenger cars, buses and trucks, are distributed randomly on the road, and their actions are generated stochastically, including lane changes, accelerating/decelerating and cruising in the scenario. The velocity of surrounding vehicles is limited below 120 km/h, and their acceleration ranges from −0.8 g to 0.5 g. The driving situations are generated before the experiment randomly but remain unchanged for different participates. Two drivers are invited to participate in the experiment, and both have held their licenses for more than three years and driven more than 1000 miles. They drive in the same situations, and no interference is permitted for the driver during the driving period. The statistical results of the driving data are shown in Figure 3. The distribution of acceleration accords approximately with the normal distribution. The distance between the lead and host vehicles (denoted by “Range” in Figure 3) is limited below 120 m to extract the car-following data.

### 3.2. ANN-NARX Model of Driver Behavior

The driver behavior model includes two functionalities, one of which is for the lateral and the other one being for the longitudinal. Wang et al. have made an analysis on the factors affecting driver lateral behavior [24]. Referring to their work, we model the lateral behavior with the same input variables, including the ego-vehicle speed, relative yaw angle, lateral distance and road curvature. The longitudinal behavior is modeled in reference to [29], whose inputs are the range, relative velocity, while the output is the velocity. In this study, the acceleration is the predicted variable, and the ego-vehicle speed and road curvature act as extra inputs to cover more complicated conditions.

The expression of NARX with an input delay to describe the reaction time of the driver is:

(4) Y(t)=f(X(t−d)…X(t−1),X(t−d)…X(t−1))

where Y is the output, X is the input and d is the input delay. Furthermore, considering the previous conclusion that driver’s response time is around 0.45-1 s [29], the input delay is set to 0.7 s. To take the advantages of both NARX and ANN, the ANN-NARX is set up to describe the driver behavior by using an ANN to fit the nonlinear relationship. The ANN consists of three layers, including input, hidden and output. The number of hidden layers and neurons in the hidden layer are set to 1 and 10, respectively. The Sigmoid function is applied to the hidden layer. The backpropagation methods are efficient to train ANN, and among these Levenberg–Marquardt is selected to train the ANN model because of its better learning performance [32]. The mean square error (MSE) is used to describe the accuracy of the model:

(5) $$\mathrm{MSE}\;=\frac{1}{n}\overset{n}{\underset{i=1}{\sum }}{\left(y_{o}-y_{p}\right)}^{2}$$

where $y_{o}$ is the original value, $y_{p}$ is the predicted value and n is the number of samples. The steering wheel angle and the acceleration are the outputs of the driver lateral and longitudinal models, respectively. The accuracy of the ANN-NARX models is shown in Table 1, from which it can be found that the ANN-NARX models can describe the lateral and longitudinal driving behavior finely. Moreover, the statistical results of the driver model using different driving data are also different because the two drivers behave differently in the same driving situation. This requires that the individual characteristics be taken into account when designing the detection algorithm, which is discussed in Section 4.2.

## 4. Degraded Domain (DD) Design

By using the driver model set up in Section 3, the correction ability of the driver can be evaluated in real time with onboard signals, as shown in Equation (3). According to the index of the driving capability defined in Section 2, the following DD is designed:

(6) $$\Omega_{lo}=\left\{\mathrm{TTCi}>\underline{\mathrm{TTCi}}\;\mathrm{and}\;\epsilon_{clo}=\left|a_{a}-a_{m}\right|>\sigma_{clo}\right\}\;\Omega_{la}=\left\{\mathrm{TLC}<\bar{\mathrm{TLC}}\;\mathrm{and}\;\epsilon_{cla}=\left|\delta_{a}-\delta_{m}\right|>\sigma_{cla}\right\}$$

where $\Omega_{lo}$ and $\Omega_{la}$ are the DD for longitudinal and lateral motion, $\underline{\mathrm{TTCi}}\;$ and $\bar{\mathrm{TLC}}\;$ are the thresholds of longitudinal and lateral driving risk, and $\sigma_{clo}$ and $\sigma_{cla}$ are the lower limits of the longitudinal and lateral correction ability respectively. The actual driver inputs, $a_{a}$ and $\delta_{a}$, are measured by the onboard sensors, and the desired inputs, $a_{m}$ and $\delta_{m}$, are predicted by the ANN-NARX models set up in Section 3.2. From the previous studies, it is known that a driver has his/her individual driving style. The designed method should not only have the ability to identify the degradation of the driving capability but should also be able to adapt to different drivers.

### 4.1. Modelling of DD Boundary

**Figure 4:**
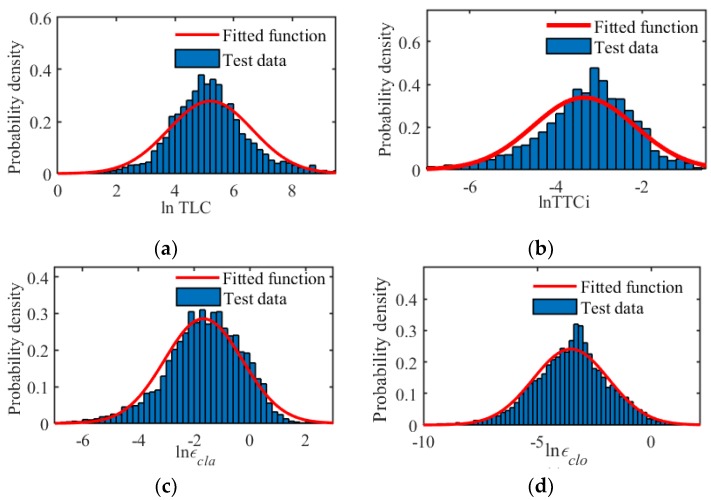
Statistical distribution of the driving risk and correction ability: (**a**) Lateral driving risk; (**b**) Longitudinal driving risk; (**c**) Lateral correction ability; and (**d**) Longitudinal correction ability.

It is best to construct the degraded domain with the driving data under both normal and abnormal conditions. Considering the fact that under abnormal or critical conditions the driver behaves more uncertainly and inconsistently, the normal driving data recorded by the driving simulator in Section 3 is used to model the boundary of DD. The statistical results about the driving risk and correction ability of driver 1 are shown in Figure 4 as an example, and that of driver 2 are similar.

From the results, it can be found that all evaluation indexes obey the log normal distribution, which provides the possibility to model and estimate the boundary of DD online. For a log normal distribution, its mean, μ, and variance, σ, can be calculated by:

(7) $$\mu =\frac{1}{N}\overset{N}{\underset{i=0}{\sum }}\ln\varepsilon_{i},\;\;\sigma^{2}=\frac{1}{N-1}\overset{N}{\underset{i=1}{\sum }}{\left(\ln\varepsilon_{i}-\mu \right)}^{2}$$

where $\varepsilon_{i}$ is the sample, and its number is N. With the parameters μ and σ, the cumulative density function (CDF), p(lnε), is:

(8) $$p=F\left(\ln\varepsilon \right)=\frac{1}{\sqrt{2\pi }\sigma }\int_{-\infty }^{\ln\varepsilon }e^{-\frac{{\left(\ln\varepsilon -\mu \right)}^{2}}{2\sigma^{2}}}/\ln\varepsilon d_{ln\varepsilon }$$

The fitted log normal distributions of the evaluation indexes are shown in Figure 4 by the red lines. Then, the boundary of DD can be calculated with the inverse CDF, $F^{-1}\left(p\right)$, and a predefined confidence value, $\bar{p}$:

(9) $$\varepsilon =e^{F^{-1}\left(\bar{p}\right)}$$

This means that the sample $\varepsilon_{i}$ is smaller than ε with the probability of $\bar{p}$. The calculation process for the boundary of different evaluation indexes is the same, and only the used sample data is different. In this paper, the confidence value is set to $\bar{p}=0.95$ for the longitudinal driving risk and longitudinal/lateral correction ability, and the confidence value of the lateral driving risk is set to $\bar{p}=0.05$ because it is known from Equation (6) that the DD of the lateral driving risk is smaller than this threshold.

### 4.2. Adaptivity to Different Drivers

From the previous studies, different drivers control the vehicle in different manners. The designed algorithm should have the ability to adapt to different drivers. Based on the boundary calculation process proposed in Section 4.1, the following algorithm is used to update the mean and variance values of CDF recursively with new samples [33]:

(10) $$\mu_{k}=\frac{\ln\varepsilon_{k-1}+N_{k-1}\mu_{k-1}}{1+N_{k-1}},\;\;\sigma_{k}^{2}=\sigma_{k-1}^{2}+\frac{N_{k-1}}{1+N_{k-1}}{\left(\ln\varepsilon_{k-1}-\mu_{k-1}\right)}^{2}$$

where k is the sample time. Compared with the algorithm using the error function, this algorithm can avoid storing a large quantity of history data and obtain the boundary value of DD directly using the new samples recursively.

## 5. Bench Test Validation and Analysis

Since it is very dangerous when the driver is distracted, the driver-in-loop simulator shown in Figure 2 is used to conduct the experimental validation. It has been demonstrated that driver inattention is one of the primary causes of crashes. Furthermore, unlike fatigue, distraction is convenient to implement. As one category of distraction, a visual-manual (VM) task brings a significant and negative effect to the driver [24]. Dealing with the texting messages on a phone is a typical one because multiple resources, including visual, manual and cognitive engagements, are required to deal with such tasks. During each experiment, the tested driver is asked to conduct 45 VM tasks in total, randomly selected from the predefined task set to ensure that the driver is unaware of future driving conditions. Since the length of distraction time is directly related to the possibility of a crash [34], the maximum time required to finish the task is designed to be 15 s. The traffic condition is the same as that in Section 3.1. However, four other drivers have been invited to conduct the tests to show the efficacy of the proposed algorithm. Before the test, the participations drive naturally without the VM task for about 20 min to be familiar with the bench and ensure the convergence of DD parameters.

### 5.1. Adaptability of DD Boundary

**Figure 5:**
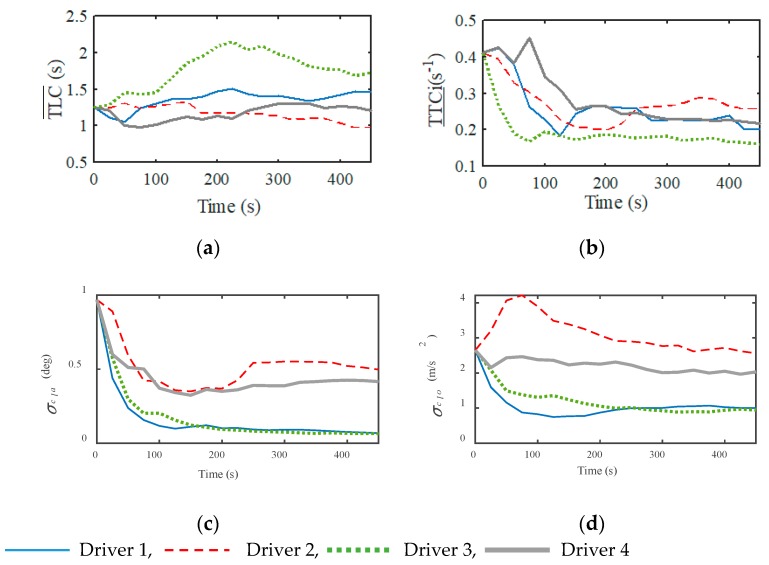
Interactive updating process of the DD boundary: (**a**) Lateral driving risk; (**b**) Longitudinal driving risk; (**c**) Lateral correction ability; and (**d**) Longitudinal correction ability.

The main motivation of this study is that assistance may not be necessary, even when the driver is inattentive, and that the capability of the driver to manage the vehicle in a risk-free state should also be considered. The main part of the proposed detection approach is DD, whose interactive updating process during the bench test is shown in Figure 5.

From the results, it is found that: (a) Though the pre-defined boundary of DD is not suitable, after about 450 s the value almost converges to a constant; (b) The DD boundaries of different drivers are different because their driving style is not the same, which is consistent with the results in Section 3.2. The results show that the proposed model of DD with its online updating algorithm described by Equations (8)–(10) converges finely, which can reflect the individual driving style. 

### 5.2. Accuracy of DD Based Detection Strategy

To evaluate the accuracy of the detection strategy based on DD, the following statistical index is defined:

(11) $$\lambda_{a}=\frac{N_{TP}}{N_{TP}+N_{FP}+N_{TN}}$$

where $\lambda_{a}$ is the accuracy, $N_{TP}$, $N_{FP}$ and $N_{TN}$ are the numbers of the true positive, false positive and true negative events, which are illustrated as the following:

True positive (TP): A TP event means that the driving state falls into DD and a real risky event happens, i.e., lane departure or rear-end crash.
False positive (FP): FP represents an event in which there is no risk but the driving state is wrongly judged to be in DD.
True negative (TN): TN is an event in which the driving state is out of the DD, but a real risk happens.

In this test, to objectively evaluate the accuracy of DD, there is no automatic control and even the driving state is judged to be in DD. The real risky event, i.e., lane departure or rear-end crash, acts as the objective index. During the whole test, there is no FP event. By further considering the driver correction ability, the unnecessary intervention is avoided successfully. There are in total 275 actual lane departure events, among which 251 are detected by DD, and the detection accuracy for lateral driving is 91.3%. The average time required by the tested drivers to correct the lane departure events (i.e., from the time when TLC starts to be smaller than zero to that when TLC becomes greater than zero) is about 1.35 s. Without cooperative assistance systems, the tested drivers generate 139 rear-end collisions, under which humans fail to detect the collision risk and drive the vehicle to a safe state. DD detects 123 rear-end collisions with an accuracy of 88.4%.

**Figure 6:**
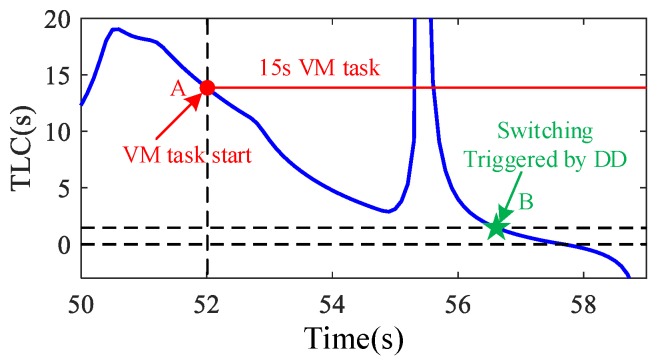
An example of the lane departure process.

A typical scenario of lane departure is selected randomly to analyze the dynamical process, as shown in Figure 6. In this scenario, the distraction task starts at about 52 s (Point A in Figure 6) and lasts for 8 s, which is a relatively long time. At 52 s, the TLC is about 14 s, at which point there is no driving risk and no assistance is required according to previous studies [8]. If the cooperation system is only designed considering the driver’s distraction, the automatic driving system is activated too early and may generate an intervention to the driver. With the DD-based switching logic, the switching time is at about 56.8 s (Point B in Figure 6), where the TLC is about 1.7 s and the condition is more dangerous. During the distraction period, a dangerous event may in practice happen at any time. Sometimes it occurs at the beginning of the distraction and sometimes near the end of the distraction. However, the time length of the distraction is hard to predict. Compared with the driver state, the cooperative driving system using a DD-based detection algorithm can avoid unnecessary interventions more effectively.

## 6. Cooperative Driving Application

**Figure 7:**
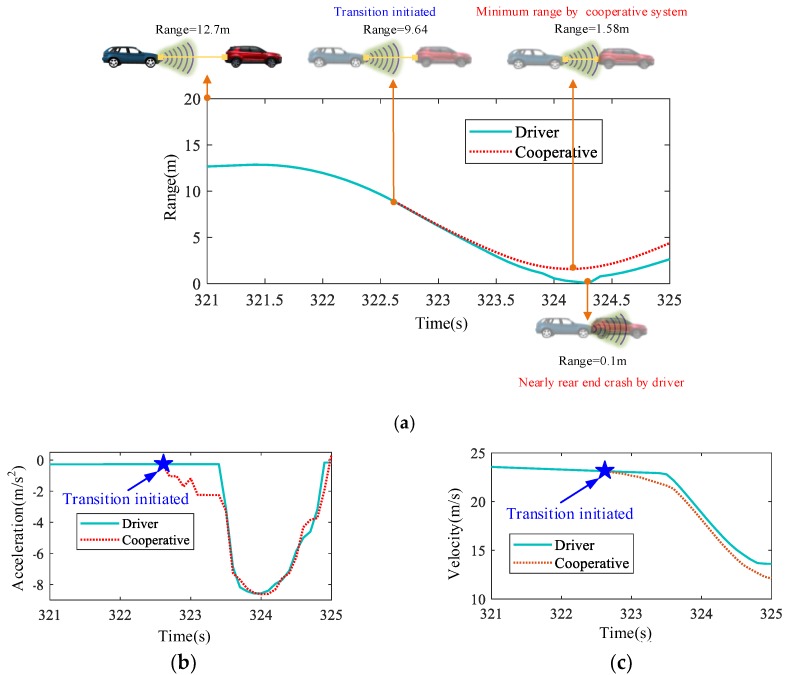
Rear-end crash corrected by the cooperative system: (**a**) Longitudinal cooperation scenario; (**b**) Acceleration profile; and (**c**) Velocity profile.

**Figure 8:**
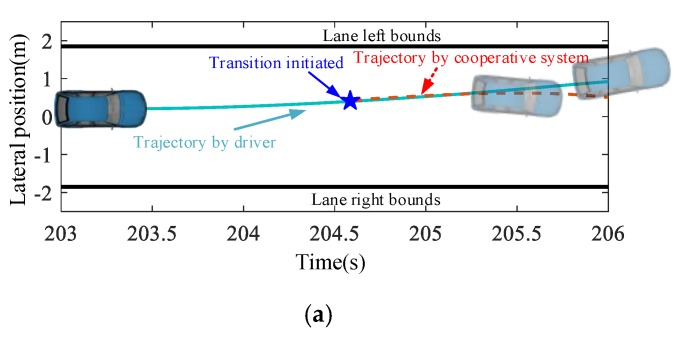

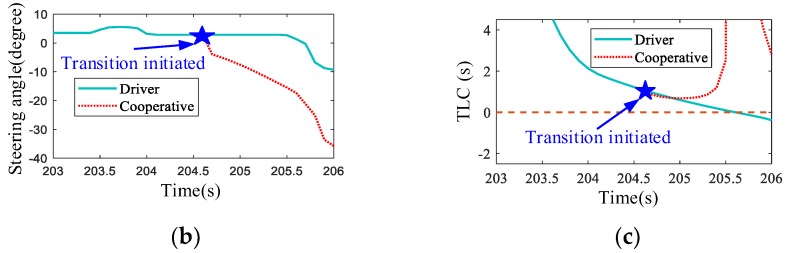
Lane departure corrected by the cooperative system: (**a**) Lateral cooperation scenario; (**b**) Steering angle profile; and (**c**) TLC profile.

From the results in the last section, it can only be concluded that the proposed evaluation index can detect the degradation of the driving capability. In this section, the performance of the complete cooperative driving process is further analyzed to show whether the DD-based detection method can reserve enough time for the automatic driving system to control the vehicle to a safe condition. This paper focuses on the design of the detection algorithm for the driving capability, and to realize a complete cooperative driving scenario the driver model learned in Section 3 acts as the control algorithm of automatic driving. Some typical scenarios are selected randomly for longitudinal and lateral cooperation, as shown in Figure 7 and Figure 8 respectively.

Figure 7 shows a near-rear-end crash scenario. If the vehicle is driven manually, the range is only 0.1 m at about 324.3 s, which is very dangerous. The proposed DD-based algorithm identifies the degradation of the driving capability at about 322.6 s and activates the automatic driving system to avoid the collision successfully. The minimum clearance between two vehicles is greater than 1.5 m, which is enough to ensure safety. From Figure 7b,c, it is found that there is no significant change in the vehicle state and that the driving authority is smoothly changed from manual to automatic.

Figure 8 shows a lane departure scenario, and the vehicle drives gradually away from its lane at about 205.6 s because of the distraction of the driver. The proposed DD-based detection algorithm successfully identifies the degradation of the driving capability and activates the automatic driving mode to correct the driving direction at about 204.6 s. After this time, the vehicle is gradually controlled to the center of the lane. Similarly to the longitudinal cooperation, the transition of the steering control from manual to automatic is smooth enough, as shown in Figure 8b. Furthermore, from Figure 8c it can be found that TLC is greater than zero during the complete cooperative driving process, which implies that the vehicle does not go to the adjacent lane.

Summarizing the cooperative validation results, it is concluded that the proposed DD-based detection strategy can reserve enough time to correct the vehicle state, besides detecting the driving capability degradation accurately. Moreover, the transition process is smooth enough to avoid a thrilling feeling for the driver.

## 7. Conclusions and Future Work

In this paper, a new evaluation index for the driving capability considering both the driving risk and correction ability of drivers is proposed. Based on this index, a DD-based detection algorithm for driving capability degradation is further designed for cooperative driving. From the bench experimental results and applications to cooperative driving, the following conclusions were obtained:

(1) The driving process is affected by many factors, such as driving risk, drive state and control ability of the driver. An evaluation index that only considers one of these will cause negative interventions for the driver. A comprehensive evaluation of driving is necessary for cooperative driving.
(2) The proposed driving capability, including both the driving risk and correction ability of drivers, can evaluate the manual driving process, and the online updating algorithm for the DD parameter is convergent and can finish the process of parameter identification within an acceptable time.
(3) The DD-based degradation detection method can be used to determine the transition time from manual driving to automatic, to reserve enough time for the automatic driving system.

The following open questions are worth investigating further:

(1) This study is based on the data acquired from a simulated driving system with four drivers. More drivers would be better, in order to show its efficacy and adaptivity.
(2) The decrease of the driving capability is simulated by VM tasks in this study, which is just one of the factors reducing the driving capability. It is better to evaluate the designed cooperative driving strategy considering more factors, such as fatigue, drugs, alcohol, and so on.

## Figures and Tables

**Table 1:** Accuracy of the Artificial Neural Network for Nonlinear Autoregressive Exogenous Process (ANN-NARX) model.

	Driver	Training MSE	Test MSE	Average MSE
Lateral [degree^2^]	1	0.189	0.186	0.188
	2	1.1516	1.018	1.112
Longitudinal [m^2^/s^4^]	1	0.0029	0.003	0.0029
	2	0.0790	0.0833	0.0803
